# Synergy of botanical drug extracts from *Dracaena cochinchinensis* stemwood and *Ardisia elliptica* fruit in multifunctional effects on neuroprotection and anti-inflammation

**DOI:** 10.3389/fphar.2024.1399549

**Published:** 2024-05-01

**Authors:** Dusadee Ospondpant, Queenie Wing Sze Lai, Tina Tingxia Dong, Karl Wah Keung Tsim

**Affiliations:** ^1^ Division of Life Science and Center for Chinese Medicine, The Hong Kong University of Science and Technology, Kowloon, Hong Kong SAR, China; ^2^ Shenzhen Key Laboratory of Edible and Medicinal Bioresources, HKUST Shenzhen Research Institute, Shenzhen, China

**Keywords:** Dracaena cochinchinensis, Ardisia elliptica, neuronal differentiation, Alzheimer’s disease, amyloid beta, anti-inflammation, botanical drug combination

## Abstract

Combination therapy is one of the promising approaches in developing therapeutics to cure complex diseases, such as Alzheimer’s disease (AD). In Thai traditional medicines, the clinical application often comprises multiple botanical drugs as a formulation. The synergistic interactions between botanical drugs in combination therapies are proposed to have several advantages, including increased therapeutic efficacy, and decreased toxicity and/or adverse effects. This study aimed to explore the therapeutic functions of a botanical hybrid preparation (BHP) of two botanical drugs within a traditional multi-herbal formulation. The synergistic actions of BHP of *Dracaena cochinchinensis* stemwood (DCS) and *Ardisia elliptica* fruit (AEF) at a specific ratio of 1:9 w/w were illustrated in neuroprotection and anti-inflammation. In cultured PC12 cells, BHP of DCS and AEF showed synergistic functions in inducing neuronal differentiation, characterized by neurofilament expression and neurite outgrowth. In addition, BHP of DCS and AEF exhibited a synergistic effect in inhibiting the aggregation of Aβ, a hallmark of AD pathology. The activated BV2 microglial cells induced by LPS were synergistically suppressed by the BHP of DCS and AEF, as evaluated by the expression of pro-inflammatory markers, including TNF-α, IL-1β, and iNOS, as well as the morphological change of microglial cells. The findings suggested that the effects of BHP of DCS and AEF were greater than individual botanical drugs in a specific ratio of 1:9 w/w to enhance neuroprotective and anti-inflammatory functions.

## 1 Introduction

Alzheimer’s disease (AD) is a progressive neurodegenerative disorder and the most common cause of dementia (World Health Organization, 2017). AD is characterized by multiple pathologies and biological defects, including accumulation of amyloid beta (Aβ), hyperphosphorylation of tau, and neuroinflammation, which contribute to neuronal cell death and cognitive impairment (Alzheimer’s Association, 2022). The current therapeutics for AD only alleviate the symptoms and cannot reverse neuronal and synaptic dysfunction in patients. Given the disease’s complexity, the present focus of therapeutic development extends beyond single-target approaches to include combination therapy with multi-target strategies (Caesar and Cech, 2019). Indeed, a therapeutic combination currently utilized for the treatment of AD involves the administration of both donepezil, an acetylcholinesterase (AChE) inhibitor, and memantine, an NMDA receptor antagonist. This combined therapy received approval from the US Food and Drug Administration in 2014, which is specifically recommended for AD patients who have achieved stabilization using donepezil and memantine treatment (Cummings et al., 2019).

Botanical medicines have a long history of usage in Thailand and other Asian countries, such as China and India, for treatment of diseases and maintenance of health. Medicinal plants are considered valuable sources of bioactive phytochemicals. Several drugs are derived from medicinal plants, such as galantamine and huperzine A (Perry and Howes, 2011). The use of Thai traditional medicine is rooted in the knowledge documented in ancient textbooks that have been passed down and developed across generations. Despite their long history of use, the precise mechanisms and specific functional roles of these traditional medicines are yet to be fully explored. Thai traditional medicine typically involves the use of multiple botanical drugs comprised in a formulation. In the notion of combined therapies, the combination of two or more botanical drugs supposes that a botanical hybrid preparation (BHP), a fixed botanical drug combination with specific chemical composition, can achieve greater activity through synergistic effects arising from distinct mechanisms (Panossian et al., 2024). The synergistic interaction between multiple botanical drugs offers several benefits over monotherapy, such as increased therapeutic efficacy, lower required dosage with equivalent efficacy, and reduced toxicity and/or adverse effects (Che, et al., 2013; Panossian et al., 2024). Among BHP, combining of two botanical drugs are the most fundamental and straightforward form of drug development (Zhou, et al., 2016).

*Dracaena* *cochinchinensis* (Lour.) S.C. Chen stemwood (DCS), known as Lakkachan or Chan Deang in Thai, has been used in Thai traditional medicine as a remedy for pyretic and inflammatory conditions (Bureau of Drug and Narcotic, 2021). Previous studies have demonstrated that DCS possesses multifunctional properties, including inhibition of Aβ aggregation, protection against Aβ fibril-induced cell death, promotion of neuronal differentiation, and suppression of neuroinflammation (Ospondpant et al., 2022; Ospondpant et al., 2023). In the Royal Medical Book of King Rama V (ตำราเวชศาสตร์ฉบับหลวง รัชกาลที่ ๕), volume II, in the section of Sabba-Lakkhana Sabba-Guna Scripture, on page 376, lines 11-13, there is a mention of a traditional formula composed of nine botanical drugs, including DCS. This formula is indicated for the treatment of *“Earth”* element abnormalities, and the alleviation of heart disorders associated with irritability and madness (Committee on Document and Archive Preparation, 1999). These clinical symptoms described in the ancient text can be interpreted as today’s symptoms of AD (Mulholland, 1979). Thus, the pharmacological efficacy of this traditional multi-herbal formulation in neuroprotective functions has to be explored. However, our findings have indicated that the multi-herbal formulation exhibited weaker efficacy, as compared to DCS alone. Here, we aim to develop a simple BHP of DCS and eight other botanical drugs, exploring their potential synergistic interactions. Among the botanical drugs tested, BHP of DCS and *Ardisia elliptica* Thunb. fruit (AEF) showed the most promising synergistic activities in promoting neuronal differentiation and inhibiting Aβ aggregation. *A. elliptica*, commonly known as Ram Yai or Pilangkasa in Thai, is used as an edible vegetable and is traditionally utilized for the treatment of diarrhea with fever (Ondee et al., 2020). Thus, BHP of DCS and AEF was chosen for further development. The optimal BHP of DCS and AEF was determined at a specific ratio of 1:9 w/w, exhibiting robust synergistic activities.

The chemical constituents from DCS, such as loureirin A, loureirin B, pterostilbene, and resveratrol, as well as AEF constituents, including embelin and syringic acid, have been reported for their biological activities. Pterostilbene and resveratrol, the well-known polyphenols, have been extensively studied for their antioxidant and anti-aging properties (Li et al., 2018). Syringic acid, a phenolic acid, has exhibited potential anti-diabetic and anti-inflammatory effects (Srinivasulu et al., 2018). However, their functional roles in BHP of DCS and AEF are unknown. Therefore, the individual and phytochemical hybrid preparation (PHP) of six phytochemicals were determined to understand their potential synergistic interactions within the BHP of DCS and AEF.

## 2 Materials and methods

### 2.1 Chemicals

Single compounds, including embelin, loureirin A, loureirin B, pterostilbene, resveratrol, and syringic acid (≥98% purity, determined by HPLC) were purchased from Chengdu Biopurify Phytochemicals Ltd. (Chengdu, China). Native mouse nerve growth factor (NGF) was obtained from Alomone Labs (Jerusalem, Israel). Dexamethasone, dimethyl sulfoxide (DMSO), lipopolysaccharide (LPS), and 3-(4,5-dimethylthiazol)-2,5-diphenyl-tetrazolium bromide (MTT) were purchased from Sigma-Aldrich (St. Louis, MO).

### 2.2 Botanical drug materials

Nine botanical drugs, composed of *Ardisia elliptica* fruit, AEF [Primulaceae; *Ardisia elliptica* Thunb.], *Baliospermum solanifolium* root, BSR [Euphorbiaceae; *Baliospermum solanifolium* (Burm.) Suresh], *Dracaena cochinchinensis* stemwood, DCS [Asparagaceae; *Dracaena cochinchinensis* (Lour.) S.C. Chen], *Mesua ferrea* flower, MFF [Calophyllaceae; *Mesua ferrea* L.], *Phyllanthus emblica* fruit, PEF [Phyllanthaceae; *Phyllanthus emblica* L.], *Terminalia bellirica* fruit, TBF [Combretaceae; *Terminalia bellirica* (Gaertn.) Roxb.], *Terminalia chebula* fruit, TCF [Combretaceae; *Terminalia chebula* Retz.], *Tarenna hoaensis* stemwood, THS [Rubiaceae; *Tarenna hoaensis* Pit.], and *Zingiber officinale* root, ZOR [Zingiberaceae; *Zingiber officinale* Roscoe] were obtained from Tha Phra Chan Herb Co., Ltd. (Bangkok, Thailand) in 2020 and authenticated by Dr. Tina Ting-Xia Dong. The voucher specimens, including AEF#2020-11-25, BSR#2020-11-25, DCS#2020-11-25, MFF#2020-11-25, PEF#2020-11-25, TBF#2020-11-25, TCF#2020-11-25, THS#2020-11-25, and ZOR#2020-11-25, were deposited at the Center for Chinese Medicine, The Hong Kong University of Science and Technology.

### 2.3 Preparation of botanical drug extracts

A traditional multi-herbal formulation is composed of an equal amount of nine botanical drugs, according to The Royal Medicinal Book of King Rama V. The aqueous extracts from BHP and individual botanical drugs were prepared as follows: the coarse powder of each botanical drug (5 g) was dissolved in 200 mL boiling water and then soaked for 2 h. The solution was centrifuged at 20,000 rpm, 4°C for 10 min. The supernatant was freeze-dried. The ethanol extracts from BHP of DCS and AEF were prepared as follows: the total coarse powder of DCS:AEF (10 g), combined in nine different ratios, including 1:9, 2:8, 3:7, 4:6, 5:5, 6:4, 7:3, 8:2, and 9:1 w/w, was extracted in 90% ethanol (400 mL), and sonicated for 20 min. BHP of DCS and AEF were extracted twice. The solution was concentrated using a rotary evaporator, and then freeze-dried. In parallel, the ethanol extract from individual botanical drugs was conducted in the same manner as the BHP of DCS and AEF at a specific ratio of 1:9 w/w. The coarse powder of DCS (1 g) or AEF (9 g) was extracted in 90% ethanol (400 mL) and then sonicated for 20 min twice. The extracting yields were approximately 7.08% for DCS:AEF (1:9), 20.58% for DCS, and 6.17% for AEF (Supplementary Table S1).

### 2.4 HPLC analysis

The botanical drug extracts (at 10 mg/mL) were analyzed by HPLC-DAD using a TC-C18 column (4.6 × 250 mm, 5 μm) (Agilent, Santa Clara, CA). The mobile phase comprised 0.2% formic acid (solvent A) and acetonitrile (solvent B). The gradient program was employed as follows: 10%-15% (B) at 0-10 min; 15%-25% (B) at 10-30 min; 25%-35% (B) at 30-55 min; 35%-40% (B) at 55-65 min; 40%-60% (B) at 65-90 min; 60%-80% (B) at 90-95 min, and 80%-100% (B) at 95-120 min. The flow rate was 1.0 mL/min. The injection volume was 5 μL. The detection wavelength was performed at 295 nm. Reference compounds, including loureirin A, loureirin B, pterostilbene, and resveratrol, were used to represent the constituents of DCS. Similarly, syringic acid and embelin were employed as reference compounds for AEF.

### 2.5 Cell cultures and cell viability

Rat pheochromocytoma PC12 cells were obtained from American Type Culture Collection (ATCC 1721, Manassas, VA). PC12 cells were cultured in DMEM supplemented with 6% fetal bovine serum (FBS), 6% horse serum, and 1% penicillin-streptomycin (10,000 U/mL). The cells were under starvation in DMEM condition in 1% FBS and 1% horse serum for 24 h before the treatment. Mouse microglial BV2 cells were purchased from National Infrastructure of Cell Line Resource (NICR, Beijing, China) with Cat# 4201MOU-CCTCC00311. BV2 cells were grown in DMEM containing 10% heat-inactivated FBS and 1% penicillin-streptomycin (10,000 U/mL). Both PC12 cells and BV2 cells were cultured until passage 20. Cell viability was determined using an MTT assay. The cultures were seeded in a 96-well plate for 24 h. BV2 cells, 2 × 10^4^ cells/well, and PC12 cells, 1 × 10^4^ cells/well, were treated with botanical drug extracts or single compounds for 24 h and 48 h, respectively. Following the treatment, MTT reagent was added to each well at a final concentration of 0.5 mg/mL. The absorbance of the formazan was measured at 570 nm.

### 2.6 DNA transfection and luciferase activity

Cultured PC12 cells were transfected with DNA, which constructed the reporter gene of pNF68-Luc or pNF200-Luc, using jetPRIME (Polyplus Transfection, New York), according to the manufacturer’s protocol. The cells were treated with botanical drug extracts (0.5-20 μg/mL) or single compounds (0.5-10 µM), with or without a low dose of NGF (1.5 ng/mL), for 24 h. The cells were lysed by passive lysis buffer (Promega, Madison, WI). Cell lysates were performed luciferase activity using a kit (Promega, Madison, WI), and normalized by protein concentration.

### 2.7 Neurite outgrowth assay

Cell differentiation was examined by the extension of neurites. PC12 cells, 3 × 10^4^ cells/well, were seeded in a 12-well plate. The cultures were treated with botanical drug extracts, with or without a low dose of NGF (1.5 ng/mL), for 48 h. The cells treated with NGF at 50 ng/mL were performed as a positive control. After the treatment, the images of cell morphology, including neurite length, were captured randomly under the phase-contrast microscope with ×10 objective lens (Carl Zeiss, Oberkochen, Germany) using SPOT imaging software (Diagnostic Instruments, MI). The length of neurites of 100 cells was measured using ImageJ software. The neurite outgrowth was characterized as follows: neurite length was less than 15 μm, 15–30 μm, and more than 30 μm.

### 2.8 Real-time qPCR analysis

The cultured PC12 cells, 1.5 × 10^5^ cells/well in a 12-well plate, were treated with botanical drug extracts (0.5-20 μg/mL), with or without NGF (1.5 ng/mL), for 24 h. NGF at 50 ng/mL was used as the positive control for stimulating neurofilament expression. The cultured BV2 cells, 1.6 × 10^5^ cells/well in a 12-well plate, were pretreated with botanical drug extracts (0.1-10 μg/mL) or single compounds (0.1-5 μM) for 4 h, and then exposed to LPS (100 ng/mL) for 20 h. Dexamethasone at 10 μM was employed as the positive control for suppressing pro-inflammatory molecule mRNA levels. Total RNA was extracted by RNAzol^®^RT (Molecular Research Center, OH), according to the manufacturer’s instruction, and converted to cDNA by PrimeScript RT Master Mix (Takara Bio, Japan). Real-time qPCR was conducted using Roche SYBR Green Master mix (Roche, Switzerland) under Roche 480 multiplex quantitative PCR system. The relative mRNA levels of target genes were quantified by the 2^−ΔΔCT^ method and normalized by GAPDH mRNA. The sequence of primers was presented in Supplementary Table S2.

### 2.9 Western blot analysis

The expression of neurofilaments was determined using Western blotting. After the treatment of PC12 cells with botanical drug extracts (1-20 μg/mL), with or without NGF (1.5 ng/mL), for 48 h, the cultures were lysed with low salt lysis buffer containing protease inhibitors, including aprotonin, leupeptin, benzamidine and pepstatin A. Cell lysates were centrifuged at 12,000 rpm for 15 min at 4°C. The supernatants were collected, and the amounts of protein were measured using the Bradford assay. Proteins were separated in 8% SDS-polyacrylamide gel electrophoresis and then transferred to nitrocellulose membranes. The membranes were blocked with 5% bovine serum albumin and probed with primary antibodies directed against neurofilament 160 (NF160) and α-tubulin (Cell Signaling Technology, Danvers, MA) at 4°C overnight. Subsequently, the membranes were incubated with secondary antibodies and exposed to an enhanced chemiluminescence (ECL) Western blotting detection kit (Thermo Fisher Scientific, Waltham, MA). The protein bands were visualized using the ChemiDoc Touch Imaging System (Bio-Rad). The intensity of protein bands was analyzed by ImageJ.

### 2.10 Immunofluorescence staining

The morphology of BV2 cells was illustrated by using immunofluorescence staining. BV2 cells, 1 × 10^5^ cells/dish, were cultured in a 35-mm^2^ confocal dish for 24 h. The cells were pretreated with botanical drug extracts at 10 μg/mL for 4 h, before treatment of 100 ng/mL LPS for 20 h. Dexamethasone at 10 μM was employed as a positive control to suppress LPS-induced inflammation. Subsequently, the cells were fixed with 4% paraformaldehyde for 20 min and blocked with 5% bovine serum albumin in PBS containing 0.2% Triton X-100 for 1 h. The cells were then incubated overnight at 4°C with an anti-Iba1 antibody (ab178846, Abcam) at a dilution of 1:500. Alexa Fluor 647-conjugated anti-rabbit antibody was used as the secondary antibody at a dilution of 1:200 and incubated for 2 h. The nuclei were stained with 4′,6-diamidino-2-phenylindole (DAPI, 1:500). The cells were visualized under a Leica SP8 confocal microscope (Wetzlar, Germany) using a ×40 oil immersion objective.

### 2.11 Preparation of Aβ_1-42_ fibrils

Purified synthetic Aβ_1-42_ (GL Biochem, Shanghai, China) was dissolved in 100% hexafluoroisopropanol and sonicated for 20 min at room temperature. The Aβ_1-42_ solution was dried out overnight in the fume hood. Aβ_1-42_ fibrils were prepared by re-suspending the dried Aβ_1-42_ peptide film (0.5 mg) with 20 μL DMSO and diluting with 10 mM HCl to a final concentration of 100 µM. The Aβ_1-42_ solution was vortexed for 1 min and incubated at 37°C for 6 days.

### 2.12 Thioflavin T fluorescence and atomic force microscopy assays

Aβ_1-42_ fibrils were determined using thioflavin T (ThT) fluorescence dye. The morphology of Aβ_1−42_ fibril or aggregate forms was visualized using atomic force microscopy (AFM). To investigate the inhibition of Aβ_1-42_ fibril formation, Aβ_1-42_ monomers (10 µM) were incubated with botanical drug extracts (0.5-20 μg/mL) or single compounds (0.5-10 μM) at 37°C for 6 days. To examine the disassembly of Aβ aggregation, Aβ_1-42_ fibrils (10 µM) were aggregated with botanical drug extracts (0.5-20 μg/mL) at 37°C for 5 days. Curcumin (30 µM) was performed as a positive control. ThT fluorescence dye was added with a final concentration of 20 µM. The intensity of ThT fluorescence was measured at an excitation wavelength of 435 nm, and emission wavelength of 488 nm, which was conducted in a 96-well black plate. The AFM images were visualized after applying the samples (30 µL) onto the mica sheets (1 × 1 cm^2^) using tapping mode.

### 2.13 Synergism analysis of combination

The synergistic effects of botanical drug combination were performed by the Chou-Talalay method (Chou, 2006) using CompuSyn software 1.0 (ComboSyn, Paramus, NJ). The combination index (CI) was identified as synergistic, additive, and antagonistic effects, where CI < 1, CI = 1, and CI > 1, respectively.

### 2.14 Statistical analysis

Data were analyzed in the form of concentration-response curve of BHP compared to individual botanical drugs and performed at least three independent experiments (*n* ≥ 3), which were presented in mean ± SEM. The statistical analysis was conducted using one-way analysis of variance (ANOVA) with Tukey’s *post hoc* test by GraphPad Prism 9.0.0. The statistical significance was defined by (*) *p* < 0.05, (**) *p* < 0.01, and (***) *p* < 0.001.

## 3 Results

### 3.1 The botanical drug combination in neuronal differentiation and Aβ fibrilization

**FIGURE 1:**
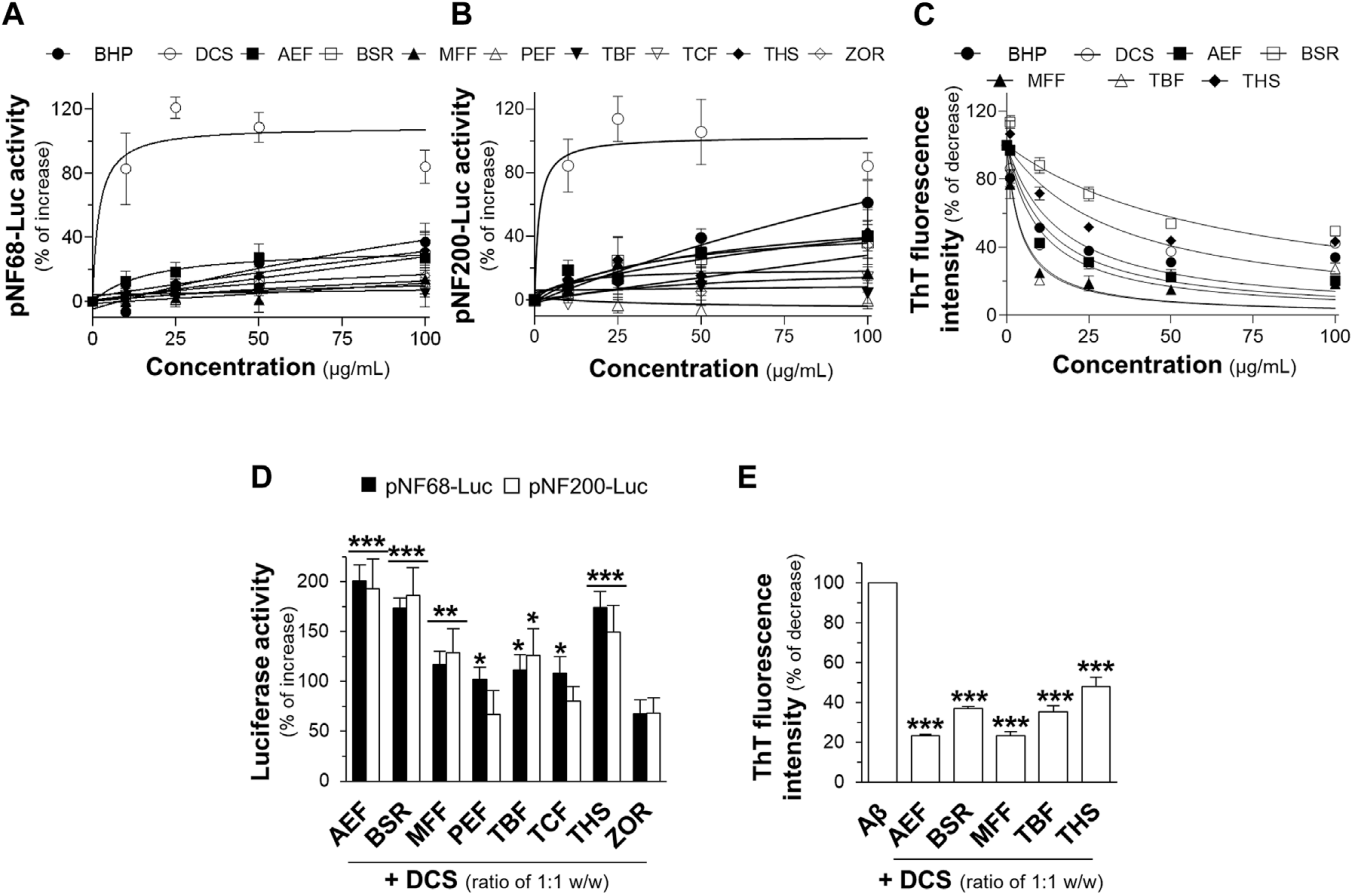
Botanical hybrid preparations (BHP) and single botanical drugs in promoting neuronal differentiation and inhibition of Aβ_1-42_ fibril formation. PC12 cells transfected with **(A)** pNF68-Luc, and **(B)** pNF200-Luc were treated with aqueous extracts from BHP of nine botanical drugs or botanical drugs, 10-100 μg/mL, for 24 h. **(C)** Co-incubation of BHP of nine botanical drugs or botanical drugs, 10-100 μg/mL, with 10 μM Aβ_1-42_ monomers for 6 days decreased the ThT fluorescence intensity in dose-dependent manners. **(D)** The aqueous extracts from BHP of DCS and other botanical drugs, with the ratio of 1:1 w/w, at 100 μg/mL increased the activities of pNF68-Luc and pNF200-Luc. **(E)** Co-incubation of BHP of DCS and other botanical drugs, 100 μg/mL, with 10 μM Aβ_1-42_ monomers reduced the intensity of ThT fluorescence dye. Data are presented in mean ± SEM of the percentage of increase or decrease, compared to the control (*n* = 4). *p < 0.05, **p < 0.01, and ***p < 0.001 as compared to the control.

Thai traditional medicines have been utilized for their therapeutic effects since ancient times. However, several traditional remedies still require identifying and verifying their therapeutic functions. In this study, the aqueous extract from a traditional multi-herbal formulation, documented in the Royal Medical Book of King Rama V, volume II, in the section of Sabba-Lakkhana Sabba-Guna Scripture, on page 376, lines 11–13, was investigated for its potential activities in promoting neuronal differentiation and reducing Aβ_1-42_ fibril formation. Neuronal differentiation is characterized by the expression of neurofilaments that play a vital role in cell differentiation and maintenance (Yuan and Nixon, 2021). Cultured PC12 cells were transfected with pNF68-Luc or pNF200-Luc, and then treated with non-toxic concentrations, 10-100 μg/mL of aqueous extracts from BHP of nine botanical drugs and individual botanical drugs (Supplementary Figure S1A). Treatment with aqueous extracts of the BHP or single botanical drugs resulted in a dose-dependent increase in luciferase activities of the reporter genes (Figures 1A, B). Among the botanical drug extracts, DCS showed a robust increase in the activities of pNF68-Luc and pNF200-Luc. The BHP and other individual botanical drugs did not show strong responses. Besides, the formation of Aβ_1-42_ fibrils and aggregates was assessed using ThT fluorescence dye (Xue et al., 2017). Co-incubation of 10 μM Aβ_1-42_ monomers with aqueous extracts from the BHP or individual botanical drugs led to a dose-dependent reduction in ThT fluorescence intensity (Figure 1C). Notably, the aqueous extracts from AEF, MFF, and TBF showed greater ability to decrease ThT fluorescence intensity, as compared to other extracts (Figure 1C). Taken together, these findings suggested that the BHP of nine botanical drugs exhibited non-synergistic effects and limited potential for neuroprotection when compared to other botanical drugs.

To investigate potential synergistic effects, the aqueous extracts from BHP of two botanical drugs were tested at a fixed combination of 1:1 w/w ratio. Previous studies have reported that DCS alone could promote neuronal differentiation and inhibit Aβ_1-42_ aggregation (Ospondpant et al., 2022). Therefore, DCS was selected as the main component to determine potential activity in BHP. The aqueous extract from BHP of two botanical drugs, including DCS:AEF, DCS:BSR, and DCS:THS at 100 μg/mL, could increase the luciferase activities of NF-68 and NF-200 promoters by 150%-200% (Figure 1D). In addition, BHP of DCS and AEF, or MFF, resulted in approximately 80% decrease in ThT fluorescence intensity (Figure 1E). Among these BHP, DCS:AEF exhibited the most promising effect, and which therefore was selected for further analysis of synergism.

### 3.2 The synergy of DCS and AEF in promoting neuronal differentiation

**FIGURE 2:**
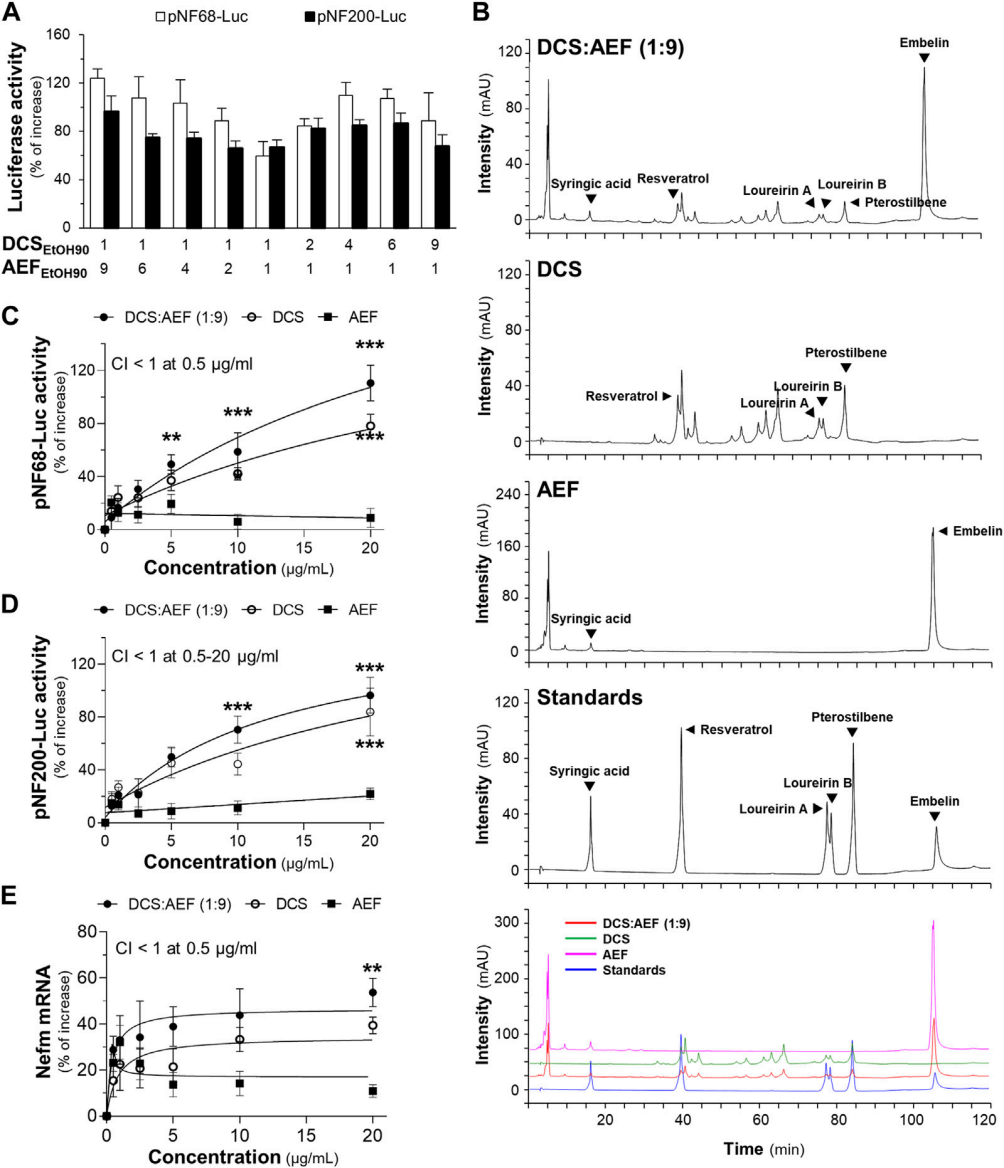
Synergistic effects of DCS:AEF on the enhancement of neuronal differentiation. **(A)** The transfected PC12 cells with pNF68-Luc or pNF200-Luc were treated with ethanol extracts from different ratios of BHP of DCS and AEF at 20 μg/mL. **(B)** The HPLC fingerprints of ethanol extracts from DCS:AEF (1:9 w/w), DCS, or AEF (at 10 mg/mL), and six reference compounds (at 1 mM) were determined using HPLC analysis with a DAD detector at the absorbance of 295 nm. The characterization of peaks in HPLC chromatograms was identified by loureirin A, loureirin B, pterostilbene, and resveratrol for DCS, as well as syringic acid and embelin for AEF. PC12 cells transfected with **(C)** pNF68-Luc, and **(D)** pNF200-Luc were treated with ethanol extracts from DCS:AEF (1:9), DCS, or AEF at 0.5-20 μg/mL for 24 h. **(E)** Nefm mRNA expression of the treatments as in **(C)**. By using CompuSyn software, where CI < 1 indicated synergism, CI = 1 indicated addition, and CI > 1 indicated antagonism. Values are shown in mean ± SEM of the percentage of increase (*n* ≥ 4). *p < 0.05, **p < 0.01, and ***p < 0.001 as compared to the control.

Previous findings have demonstrated that the ethanol extract of DCS exhibited a higher yield and greater pharmacological activities, as compared to the water extract (Ospondpant et al., 2022; Ospondpant et al., 2023). To develop a promising BHP of DCS and AEF, various ratios of DCS and AEF ethanol extracts were conducted to determine the optimal level of synergism. The BHP of DCS:AEF at the specific ratio of 1:9 w/w showed higher luciferase activities of neurofilament promoters, approximately 100%-120% increase, as compared to other ratios (Figure 2A). Consequently, the ethanol extract from BHP of DCS and AEF at 1:9 w/w ratio, denoted as DCS:AEF (1:9), was chosen for further investigation of its synergistic interactions in promoting neuronal differentiation, inhibiting Aβ aggregation, and suppressing neuroinflammation. First, HPLC analysis was performed to characterize the ethanol extracts of DCS:AEF (1:9), DCS, and AEF, and the HPLC fingerprints of these extracts were illustrated in Figure 2B. Pterostilbene and embelin were identified as the predominant peaks in DCS and AEF extracts, respectively. The contents of identified compounds in DCS:AEF (1:9), DCS, and AEF were determined using the HPLC method, as shown in Supplementary Table S1. The extract of DCS:AEF (1:9) contained approximately 5.56 μg/mg of pterostilbene and 73.08 μg/mg of embelin. The cytotoxicity assessment on BV2 cells and PC12 cells revealed that these botanical drug extracts had non-toxic concentrations of up to 10 μg/mL and 20 μg/mL, respectively (Supplementary Figures S1B, C), which were employed for subsequent analysis.

The treatment of PC12 cells with DCS:AEF (1:9) or DCS showed a significant increase in pNF68-Luc and pNF200-Luc activities (Figures 2C, D), as well as an upregulation of neurofilament medium chain (Nefm) mRNA level (Figure 2E), in a dose-dependent manner. In parallel, AEF induced a slight increase in luciferase activities and mRNA expression (Figures 2C–E). The combination of DCS and AEF, DCS:AEF (1:9) at 0.5-20 μg/mL, exhibited a synergistic effect in enhancing pNF200-Luc activity, as indicated by the CI value less than 1 (Figure 2D). However, only DCS:AEF (1:9) at 0.5 μg/mL demonstrated synergistic activities in increasing luciferase activity of NF68 promoter and mRNA expression of Nefm, as indicated by the CI value less than 1 (Figures 2C–E).

**FIGURE 3:**
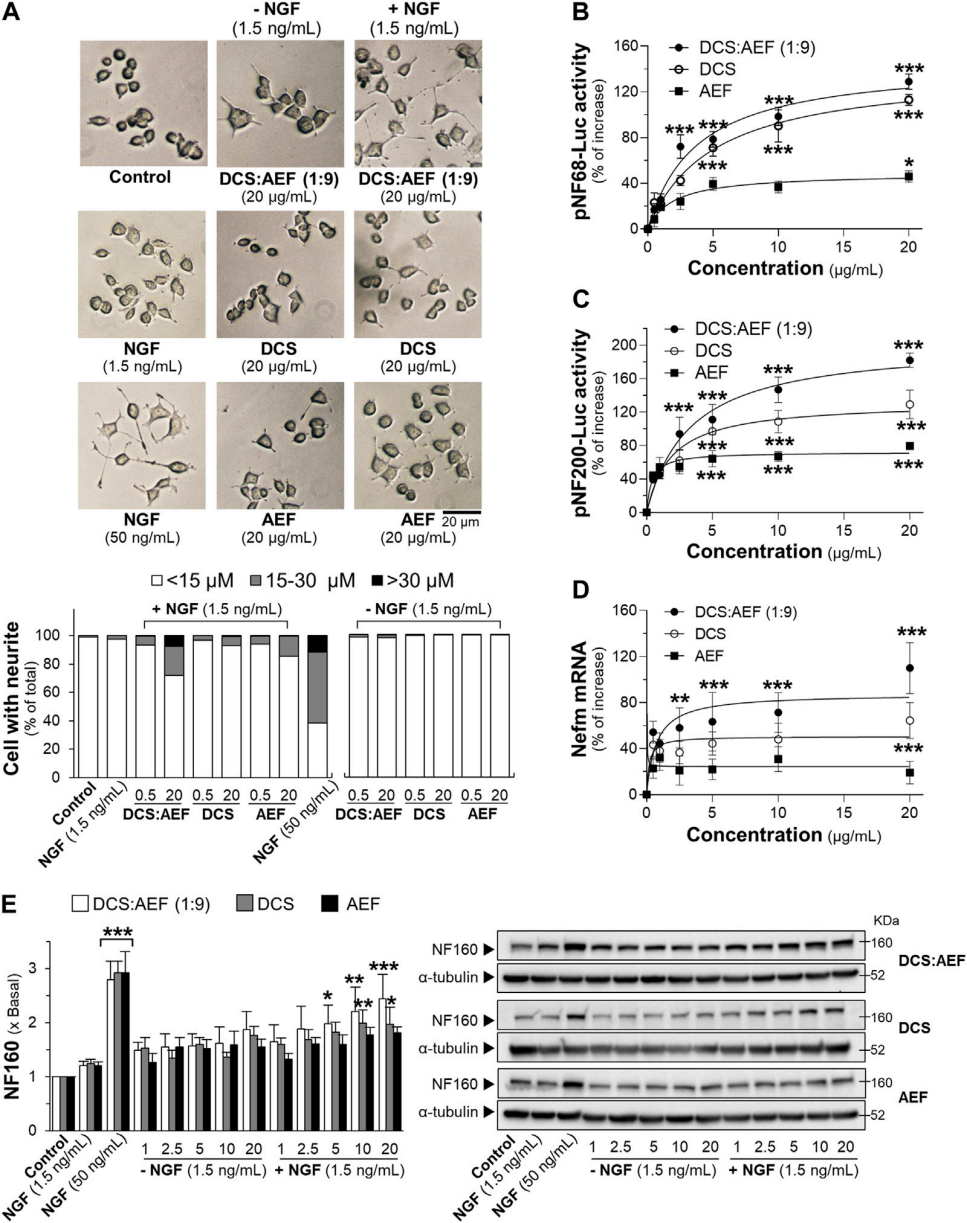
Combined effects of a low dose of NGF and DCS:AEF on stimulation of neuronal differentiation. **(A)** PC12 cells were treated with DCS:AEF (1:9), DCS, or AEF, with or without 1.5 ng/mL NGF, for 48 h, and then the neurite length was observed under a phase-contrast microscope (scale bar = 20 μm). The transfected PC12 cells with **(B)** pNF68-Luc, or **(C)** pNF200-Luc were co-treated with NGF (1.5 ng/mL) and DCS:AEF (1:9), DCS, or AEF (0.5-20 μg/mL) for 24 h. **(D)** The Nefm mRNA expression was determined after the co-treatment as in **(B)**. **(E)** Having the treatments as in **(B)**, the NF160 protein expression was determined using Western blotting. NGF at 50 ng/mL was used as a positive control for promoting neuronal differentiation. Values are shown in mean ± SEM of the percentage of increase, or fold of change (*n* ≥ 3). *p < 0.05, **p < 0.01, and ***p < 0.001 as compared to untreated cells (control).

Neurotrophic factors, such as nerve growth factor (NGF), play a crucial role in enhancing neuronal survival, development, and differentiation. The level of NGF was decreased during aging and neurodegenerative diseases (Connor and Dragunow, 1998). Here, the combined effect of a low dose of NGF (1.5 ng/mL) and botanical drug extracts on neuronal differentiation, using PC12 cells as a model system, was determined. Treatment with DCS:AEF (1:9), DCS, AEF, or 1.5 ng/mL NGF alone did not significantly increase the length of neurites (Figure 3A). However, DCS:AEF (1:9) at 20 μg/mL combined with NGF at 1.5 ng/mL showed a significant extension in neurite length (Figure 3A). In addition, the combination of DCS:AEF (1:9) and 1.5 ng/mL NGF led to an increase in luciferase activities of NF68 and NF200 promoters in dose-dependent manners (Figures 3B, C). The co-treatment of DCS:AEF (1:9) and 1.5 ng/mL NGF also increased Nefm mRNA level (Figure 3D). The protein expression of neurofilament 160 (NF160) was determined using Western blot analysis. The co-treatment of DCS:AEF (1:9), 5-20 μg/mL, and 1.5 ng/mL NGF significantly upregulated NF160 protein level by approximately 2-fold (Figure 3E).

### 3.3 The synergy of DCS and AEF in reducing Aβ_1-42_ aggregation, Aβ_1-42_ fibril-induced cell death, and inflammation

**FIGURE 4:**
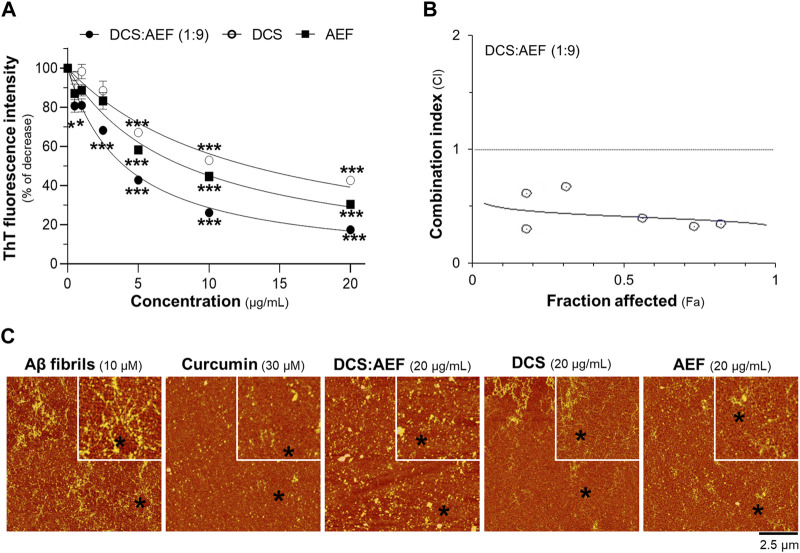
Synergistic effect of DCS:AEF on reduction of Aβ_1-42_ fibril formation. Aβ_1-42_ monomers (10 µM) were incubated with DCS:AEF (1:9), DCS, or AEF (0.5-20 μg/mL) for 6 days. **(A)** Aβ_1-42_ fibrils were determined using ThT fluorescence assay. **(B)** CI values were plotted against Fa values of ThT fluorescence intensity, using CompuSyn software, where CI < 1 indicated synergism, CI = 1 indicated addition, and CI > 1 indicated antagonism. **(C)** The morphology of Aβ_1-42_ fibrils was illustrated under the AFM with a scan size of 10 µm. Curcumin (30 µM) was used as positive control inhibiting Aβ_1-42_ fibrilization. Data are presented in mean ± SEM of the percentage of decrease (*n* = 4). *p < 0.05, **p < 0.01, and ***p < 0.001 as compared to the group of Aβ_1-42_ only (control).

To determine the inhibitory effect of botanical drug extracts on Aβ_1-42_ fibril formation, DCS:AEF (1:9), DCS, or AEF, was co-incubated with 10 μM Aβ_1-42_ monomers for 6 days. DCS:AEF (1:9) significantly reduced the intensity of ThT fluorescence by 20%-80%, in a concentration-dependent response (Figure 4A). Similarly, DCS and AEF individually showed significant decreases in ThT fluorescence intensities by 30%-60% and 40%-70%, respectively (Figure 4A). Notably, BHP of DCS and AEF exhibited a stronger effect in blocking Aβ_1-42_ fibril formation, as indicated by the CI value less than 1 (Figure 4B). The morphology of Aβ_1-42_ fibrils was observed under AFM with a scan size of 10 μm. After co-incubation with botanical drug extracts, the formation of Aβ_1-42_ fibrils was visibly reduced (Figure 4C).

**FIGURE 5:**
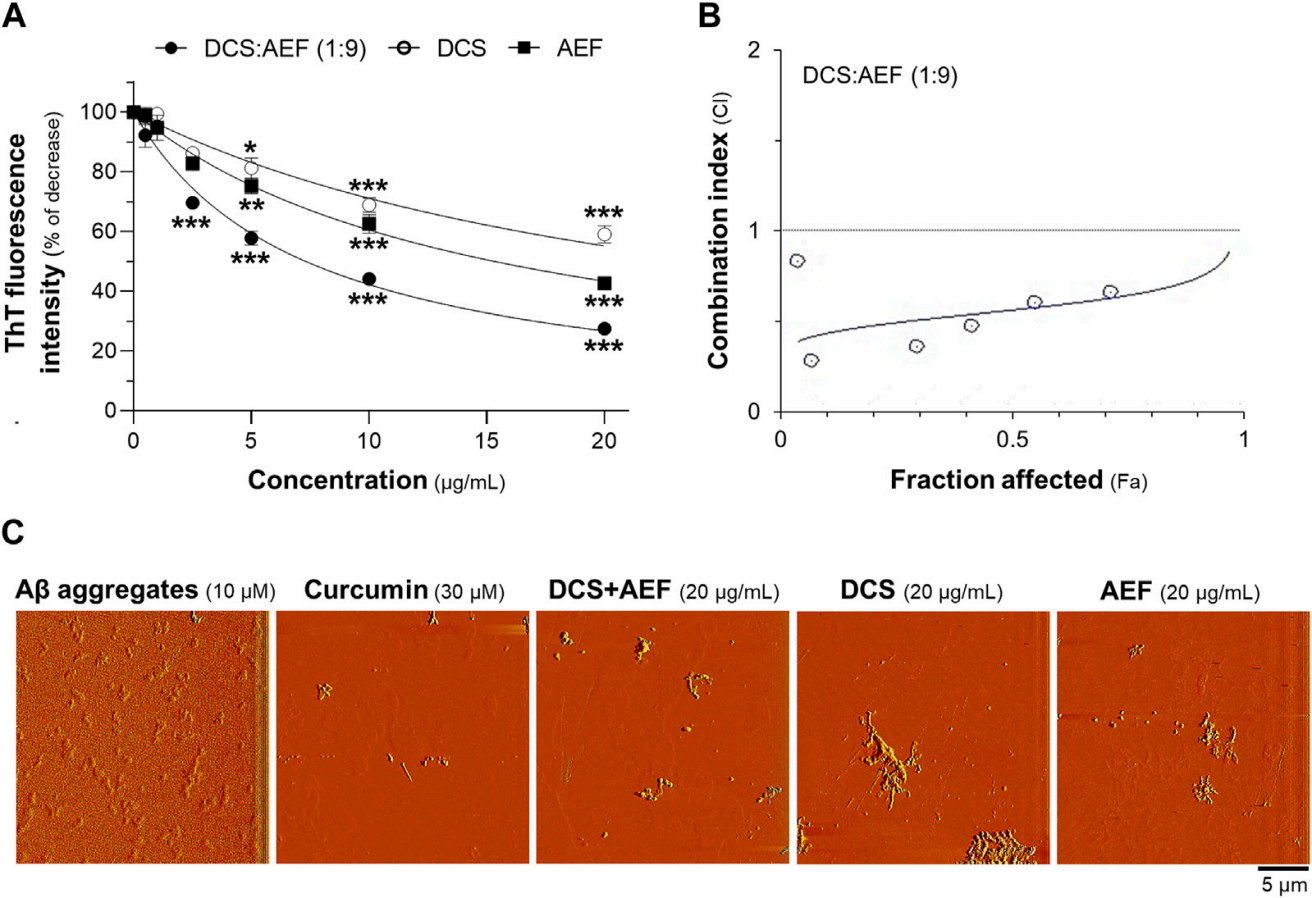
Synergistic effect of DCS:AEF on disassembly of Aβ_1-42_ aggregation. Aβ_1-42_ fibrils (10 µM) were incubated with DCS:AEF, DCS (1:9), or AEF (0.5-20 μg/mL) for 5 days. **(A)** Aβ_1-42_ aggregates were determined using ThT fluorescence method. **(B)** The plot of CI values versus Fa values of ThT fluorescence intensity was generated using CompuSyn software, where CI < 1 indicated synergism, CI = 1 indicated addition, and CI > 1 indicated antagonism. **(C)** The morphology of Aβ_1-42_ aggregates was captured under the AFM with a scan size of 20 µm. Curcumin (30 µM) was used as positive control inhibiting Aβ_1-42_ aggregation. Data are shown in mean ± SEM of the percentage of decrease (*n* = 4). *p < 0.05, **p < 0.01, and ***p < 0.001 as compared to the group of Aβ_1-42_ only (control).

To examine the disassembly of Aβ_1-42_ aggregates, the botanical drug extracts were co-incubated with 10 μM Aβ_1-42_ fibrils for 5 days. DCS:AEF (1:9), DCS, and AEF showed dose-dependent reductions in ThT fluorescence intensity, indicating the disruption of Aβ_1-42_ aggregation (Figure 5A). DCS:AEF (1:9) significantly decreased ThT fluorescence intensity by 30%-70%, while DCS and AEF reduced ThT fluorescence intensity by 30%-40%, and 25%-60%, respectively (Figure 5A). The synergistic activity of DCS:AEF (1:9) in disrupting Aβ_1-42_ aggregation was observed, as indicated by the CI value less than 1 (Figure 5B). AFM analysis further confirmed the reduction of Aβ_1-42_ aggregates upon co-application of the botanical drug extracts (Figure 5C).

**FIGURE 6:**
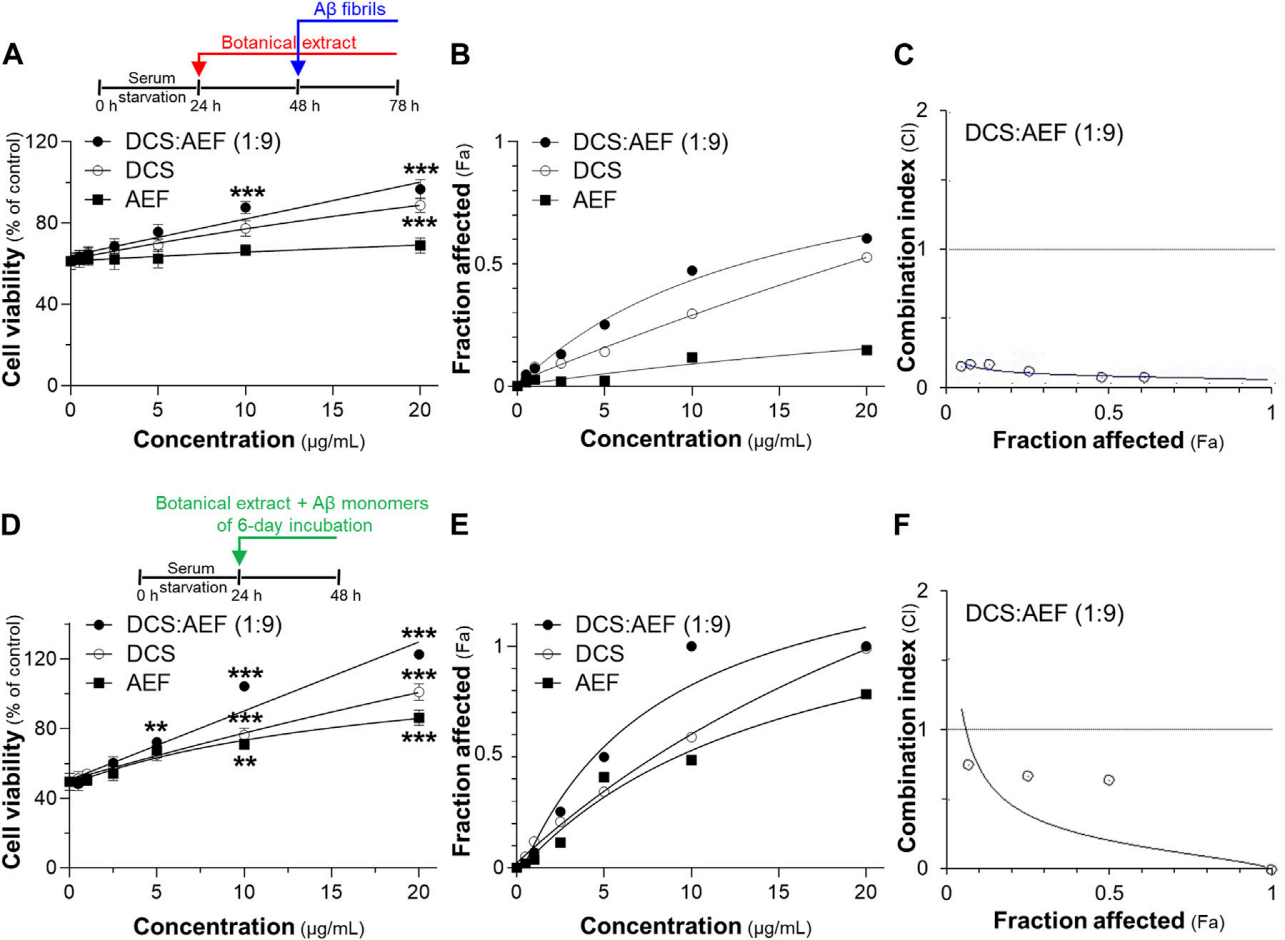
Synergistic effects of DCS:AEF on protection of Aβ_1-42_ induced cell toxicity. Cell viability was determined using an MTT assay. **(A)** The pretreatment of PC12 cells with DCS:AEF (1:9), DCS, or AEF (0.5-20 μg/mL) for 24 h before exposure to Aβ_1-42_ fibrils (10 µM) for 24. **(D)** Aβ_1-42_ monomers (10 µM) were co-incubated with DCS:AEF (1:9), DCS, or AEF (0.5-20 μg/mL) for 6 days before exposure to PC12 cells for 24 h **(B)** and **(E)** Concentration-effect curves of cell viability of **(A,D)**, respectively, were generated using CompuSyn software. **(C,F)** CI values plotted against Fa values of **(B,E)**, respectively, were analyzed using CompuSyn software, where CI < 1 indicated synergism, CI = 1 indicated addition, and CI > 1 indicated antagonism. Values are presented in mean ± SEM of the percentage of decrease (*n* = 4). *p < 0.05, **p < 0.01, and ***p < 0.001 as compared to the group of Aβ_1-42_ only (control).

Subsequently, the impact of Aβ_1-42_ fibrils on cell viability was investigated in cultured PC12 cells using two different testing designs. First, cultured PC12 cells were pretreated with botanical drug extracts before exposure to Aβ_1-42_ fibrils. Treatment with 10 μM Aβ_1-42_ fibrils alone resulted in approximately 40% loss of cell viability (Figure 6A). Notably, DCS:AEF (1:9) and DCS significantly decreased the Aβ_1-42_ fibril-induced cell death by 20%-60%, and 30%-50%, respectively, in dose-dependent responses (Figures 6A, B). However, AEF did not show a significant increase in cell viability in the presence of Aβ_1-42_ fibrils. The BHP of DCS and AEF at 0.5-20 μg/mL exhibited a strong effect in decreasing Aβ_1-42_ fibril-induced cell death, as indicated by the CI value less than 1 (Figure 6C). In the second testing method, co-incubation of 10 μM Aβ_1-42_ monomers with DCS:AEF (1:9), DCS, or AEF for 6 days before cell treatment significantly reduced Aβ_1-42_ fibril-induced cell death in dose-dependent manner, as compared to treatment with Aβ_1-42_ fibrils alone (Figures 6D, E). This finding suggested that the inhibitory effect of the botanical drug extracts on Aβ_1-42_ fibril formation was associated with the prevention of Aβ_1-42_ fibril-induced loss of cell viability. DCS:AEF (1:9) at 10-20 μg/mL completely restored cell viability in the presence of Aβ_1-42_ (Figure 6D). The synergistic effect of DCS:AEF (1:9) in reducing Aβ_1-42_ fibril-induced cell toxicity was indicated by the CI value less than 1 (Figure 6F).

**FIGURE 7:**
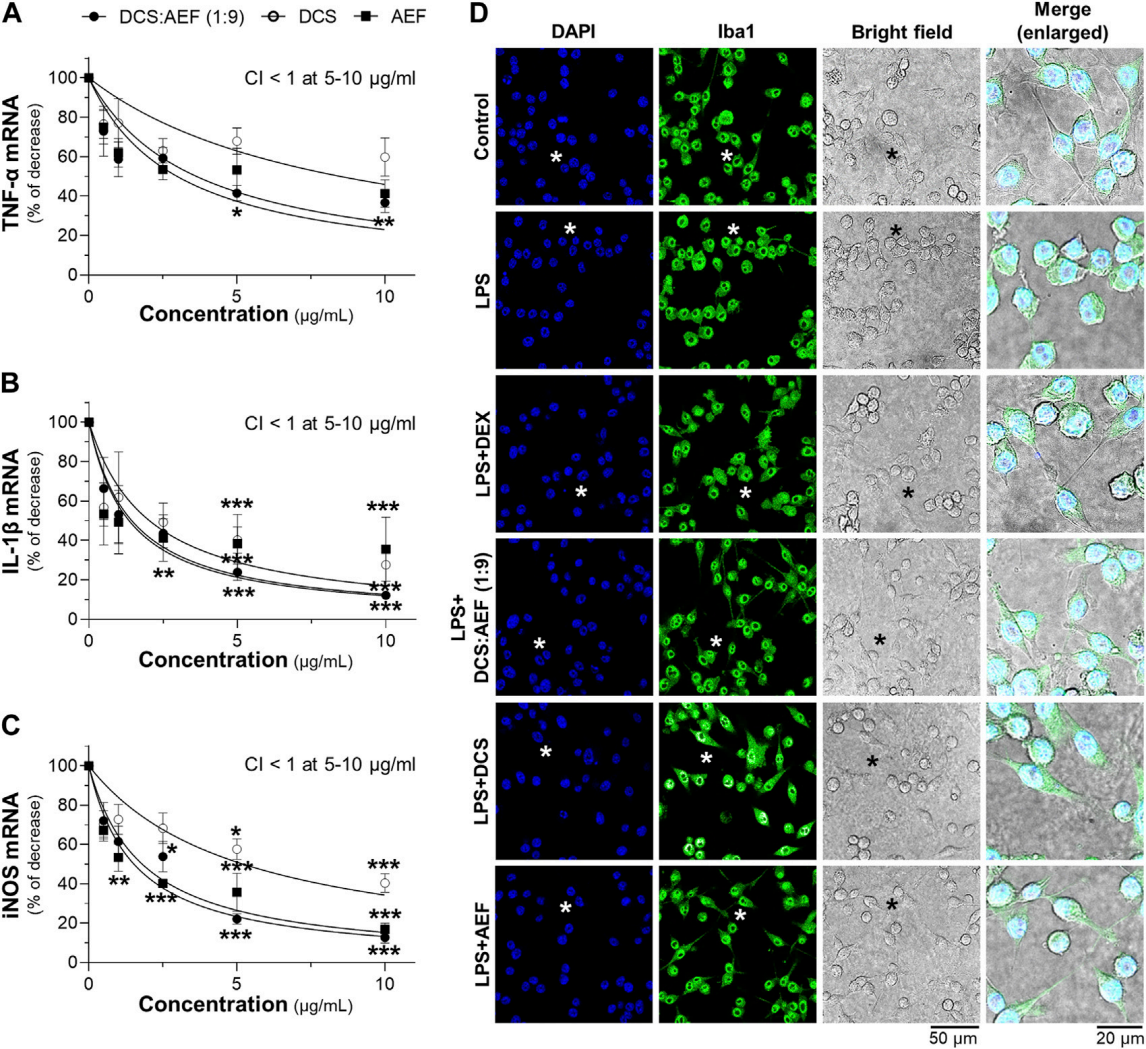
Synergistic effects of DCS:AEF on suppression of LPS-induced inflammation in microglia. BV2 cells were pretreated with DCS:AEF (1:9), DCS, or AEF (0.5-10 μg/mL) for 4 h before exposure to LPS (100 ng/mL) for 20 h. The mRNA levels of **(A)** TNF-α, **(B)** IL-1β, and **(C)** iNOS were determined using RT-qPCR analysis. Using CompuSyn software, where CI < 1 indicated synergism, CI = 1 indicated addition, and CI > 1 indicated antagonism. **(D)** The morphological changes of BV2 cells were visualized using immunofluorescence staining, under the SP8 microscope with ×40 objective. BV2 microglial cells were identified by Iba1 (green), and nuclei were detected by DAPI (blue). The asterisk indicates the enlarged area. Values are shown in mean ± SEM of the percentage of decrease (*n* ≥ 4). *p < 0.05, **p < 0.01, and ***p < 0.001 as compared to cells treated with LPS only (control).

The synergistic effect of DCS:AEF (1:9) in suppressing inflammation was determined by analyzing the mRNA expressions of pro-inflammatory markers, including TNF-α, IL-1β, and iNOS, using RT-qPCR. Treatment of BV2 cells with DCS:AEF (1:9), DCS, or AEF significantly decreased the LPS-induced mRNA expressions of TNF-α, IL-1β, and iNOS in dose-dependent manners (Figures 7A–C). DCS:AEF (1:9) showed a significant reduction of TNF-α, IL-1β, and iNOS mRNA levels by 25%-60%, 30%-85%, and 30%-80%, respectively. The suppression of LPS-induced TNF-α, IL-1β, and iNOS mRNA expressions demonstrated the synergistic interaction between the BHP of DCS and AEF at 5-10 μg/mL, as indicated by the CI value less than 1 (Figures 7B, C). In addition, the morphological changes, induced by inflammation, were examined in BV2 microglial cells. LPS-treated BV2 cells displayed roundish or amoeboid-like shapes with fewer and shorter branches (Figure 7D). However, the treatments with DCS:AEF (1:9), DCS, or AEF at 10 μg/mL, as well as dexamethasone (DEX, 10 μM), resulted in cells presenting spindle shapes, with long thin processes extending from the cell body, that was resembling the morphology of untreated cells (Figure 7D).

### 3.4 Functional roles of phytochemicals in botanical drug extracts

**FIGURE 8:**
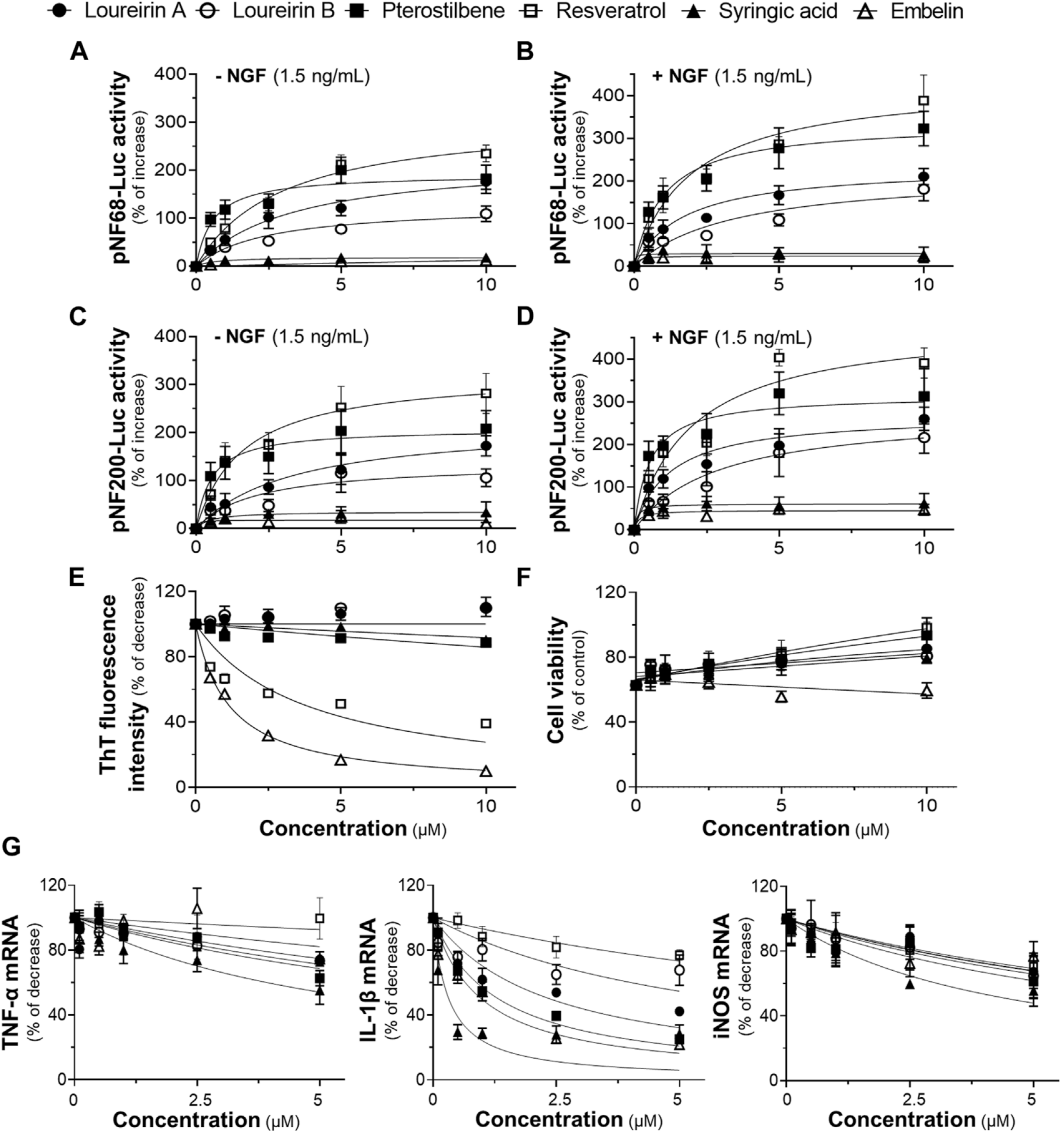
The functional roles of phytochemicals of DCS:AEF. PC12 cells transfected with **(A,B)** pNF68-Luc, or **(C,D)** pNF200-Luc were treated with single compounds, including loureirin A, loureirin B, pterostilbene, resveratrol, syringic acid, or embelin, at 0.5-10 μM, with or without NGF (1.5 ng/mL), for 24 h, and then luciferase activity was determined. **(E)** The phytochemical compound (0.5-10 μg/mL) was co-incubated with Aβ_1-42_ monomers (10 µM) for 6 days. The inhibitory effect of these compounds on Aβ_1-42_ fibril formation was determined using ThT fluorescence assay. **(F)** PC12 cells were pretreated with single compounds for 24 h before exposure to Aβ_1-42_ fibrils (10 µM) for 24 h. Cell viability was investigated using an MTT assay. **(G)** Pretreatment of BV2 cells with loureirin A, loureirin B, pterostilbene, resveratrol, syringic acid, or embelin, at 0.1-5 μM, for 4 h before exposure to LPS (100 ng/mL) for 24 h. The inhibitory effects of these compounds on TNF-α, IL-1β, and iNOS mRNA expressions were analyzed using RT-qPCR. Values are shown in mean ± SEM of the percentage of increase, and decrease (*n* ≥ 4).

The toxicity of six compounds presented in the BHP of DCS and AEF, namely, loureirin A, loureirin B, resveratrol, pterostilbene, embelin, and syringic acid, was determined in cultured BV2 cells and PC12 cells. Non-toxic concentrations of these compounds, up to 5 μM for BV2 cells, and 10 μM for PC12 cells (Supplementary Figures S1D, E), were employed to determine their activities in promoting neuronal differentiation, inhibiting Aβ_1-42_ fibril formation, preventing Aβ_1-42_ fibril-induced cell toxicity, and suppressing LPS-induced pro-inflammatory molecule expression. Treatment of PC12 cells with loureirin A, loureirin B, resveratrol, and pterostilbene showed a significant increase in luciferase activities of NF68 and NF200 promoters, in dose-dependent responses (Figures 8A, C). In parallel, when these compounds were combined with a low dose of NGF at 1.5 ng/mL, the induction of pNF68-Luc and pNF200-Luc activities was higher by 50%-100% (Figures 8B, D). Treatment of cells with embelin and syringic acid, with or without 1.5 ng/mL NGF, did not show a significant increase in pNF68-Luc and pNF200-Luc activities.

The co-incubation of Aβ_1-42_ monomers with resveratrol or embelin, at 0.5-10 μM, significantly reduced ThT fluorescence intensity by 20%-60%, and 25%-90%, respectively, in dose-dependent manners (Figure 8E). In contrast, loureirin A, loureirin B, pterostilbene and syringic acid showed non-significant decrease in ThT fluorescence intensities. Treatment of PC12 cells with loureirin A, resveratrol and pterostilbene significantly decreased Aβ_1-42_ fibril-induced cell toxicity by 40%, 40%-60%, and 30%-60%, respectively, in dose-dependent manners (Figure 8F). However, loureirin B, embelin and syringic acid did not significantly restore cell viability in the presence of Aβ_1-42_ fibrils (Figure 8F).

In cultured BV2 cells treated with 100 ng/mL LPS, loureirin A, loureirin B, pterostilbene, embelin, and syringic acid, at 5 μM, showed a significant reduction in TNF-α mRNA levels by 25%-45%. These compounds, at 0.5-5 μM, significantly decreased the LPS-induced IL-1β mRNA levels by 25%-70%. Notably, syringic acid exhibited the most potent effect in reducing IL-1β mRNA expressions. Treatment with these compounds, at 5 μM, also significantly decreased iNOS mRNA expressions by 30%-40% (Figure 8G). Resveratrol showed slight inhibitory effects on the suppression of LPS-induced mRNA expression of pro-inflammatory molecules (Figure 8G).

### 3.5 The effects of phytochemical hybrid preparation mimicking in the BHP of DCS and AEF

**FIGURE 9:**
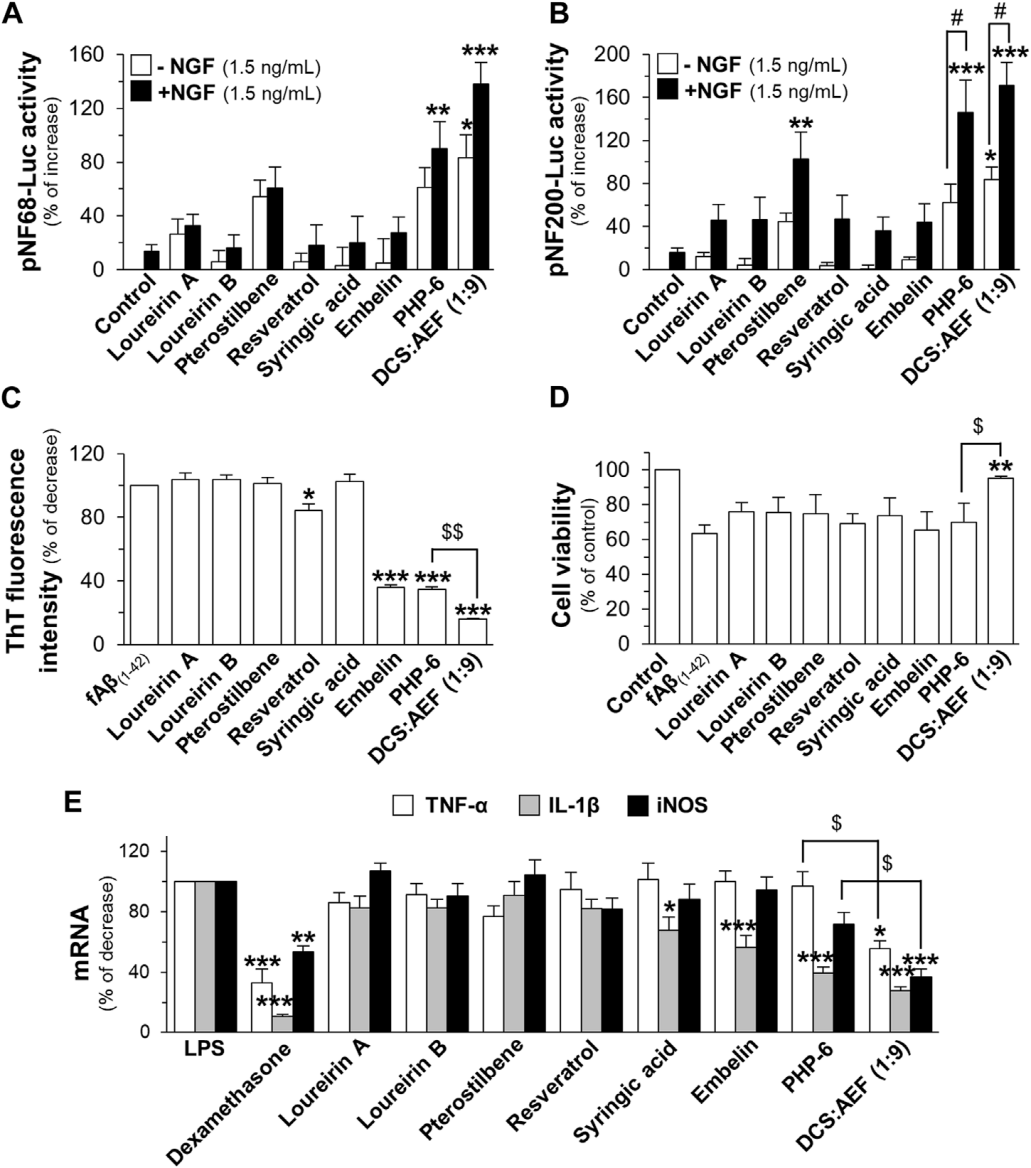
Effects of PHP of six phytochemicals mimicking the DCS:AEF. PC12 cells transfected with **(A)** pNF68-Luc, or **(B)** pNF200 Luc were treated with single compounds, PHP of six phytochemicals (PHP-6), or DCS:AEF (20 μg/mL), with or without NGF (1.5 ng/mL), for 24 h. The mimicking effects of DCS:AEF (1:9) at 20 μg/mL containing loureirin A (0.16 µM), loureirin B (0.18 µM), pterostilbene (0.42 µM), resveratrol (0.16 µM), syringic acid (0.39 µM), and embelin (4.96 µM) were determined using luciferase assay. **(C)** ThT fluorescence assay was performed on Aβ_1-42_ monomers (10 µM) co-incubated with compounds as shown in **(A)** for 6 days. **(D)** PC12 cells were pretreated with compounds as in **(A)** for 24 h, before exposure to Aβ_1-42_ fibrils (10 µM), and then analyzed by MTT assay. **(E)** BV2 cells were pretreated with loureirin A (0.04 µM), loureirin B (0.045 µM), resveratrol (0.04 µM), pterostilbene (0.11 µM), embelin (1.24 µM), syringic acid (0.98 µM), or PHP-6, replicating DCS:AEF (1:9) at 5 μg/mL, for 4 h before exposure to LPS (100 ng/mL) for 20 h. Dexamethasone (DEX, 10 µM) was employed as a positive control of anti-inflammation. TNF-α, IL-1β, and iNOS mRNA levels were determined using RT-qPCR. Data are shown in mean ± SEM of the percentage of increase, and decrease (*n* ≥ 4). *p < 0.05, **p < 0.01, and ***p < 0.001 as compared to the group of untreated cells, Aβ_1-42_ alone, Aβ_1-42_ fibril-treated cells, or LPS-treated cells. ^#^ *p* < 0.05 as compared to the group of co-treatment of 1.5 ng/mL NGF. ^$^ *p* < 0.05 as compared to the group of DCS:AEF treatment.

To determine the effects of PHP mimicking the composition of DCS:AEF (1:9), assays were conducted following a similar approach as with botanical drug extracts. PHP was composed of six phytochemicals, including loureirin A, loureirin B, resveratrol, pterostilbene, embelin, and syringic acid, denoted as PHP-6. The content of PHP-6, accounted for in the BHP of DCS and AEF at 20 μg/mL, contained 0.16 µM loureirin A, 0.18 µM loureirin B, 0.16 µM resveratrol, 0.42 µM pterostilbene, 4.96 µM embelin, and 0.39 µM syringic acid, were determined using HPLC analysis (Supplementary Table S1). In PC12 cells transfected with pNF68-Luc or pNF200-Luc, the treatments of DCS:AEF (1:9), PHP-6, and pterostilbene exhibited an enhancement of luciferase activities (Figures 9A, B). However, the other phytochemicals did not induce luciferase activities, with or without a low dose of NGF. These results suggested that pterostilbene, at least partly, might mediate the activation of neurofilaments. In addition, the co-incubation of 10 μM Aβ_1-42_ monomers with embelin, PHP-6, or DCS:AEF (1:9) significantly decreased ThT fluorescence intensity (Figure 9C). Embelin and PHP-6 exhibited similar activities in reducing ThT fluorescence intensity by approximately 65%, suggesting that the presence of embelin might predominantly contribute to the effect observed in PHP-6. However, DCS:AEF (1:9) showed greater inhibitory activity compared to PHP-6 (Figure 9C). DCS:AEF (1:9) significantly reduced cell loss induced by 10 μM Aβ_1-42_ fibrils, while individual chemicals or the PHP-6 did not show a significant increase in cell survival (Figure 9D). Next, to examine the anti-inflammatory properties of PHP-6, a combination of 0.04 µM loureirin A, 0.045 µM loureirin B, 0.04 µM resveratrol, 0.11 µM pterostilbene, 1.24 µM embelin and 0.98 µM syringic acid, was replicated in DCS:AEF (1:9) at 5 μg/mL. In the presence of LPS, the treatment with DCS:AEF (1:9) showed a greater decrease in mRNA expressions of TNF-α, IL-1β, and iNOS compared to PHP-6 (Figure 9E).

## 4 Discussion

For decades, there has been an increasing acknowledgment of the complicated nature of AD. It is widely recognized that drug monotherapy is insufficient to achieve a complete cure for AD. Therefore, the development of effective therapeutic strategies, such as the use of drug combinations, is needed. Traditional medicines, including those from Thai traditional medicine, frequently utilize multiple botanical drugs as a formulation (Committee on Document and Archive Preparation, 1999). This long-standing practice has been based on the belief that these botanical drug combinations can generate synergistic effects, leading to enhanced therapeutic efficacy and potentially reduced toxicity (Mulholland, 1979; Zhou et al., 2016). However, the scientific evidence supporting their traditional therapeutic properties is currently inadequate. In this study, BHP of nine botanical drugs was evaluated for its effects on neuronal differentiation and inhibition of Aβ fibrilization. By comparing the efficacy of BHP of nine botanical drugs to that of the individual botanical drugs, the stimulatory effect on neuronal differentiation and inhibitory effect on Aβ fibril formation have not been optimized. These findings suggested that the effects of a botanical drug might be counteracted or diminished by another botanical drug within specific targets of action (Che et al., 2013).

In BHP, combining two botanical drugs is the most basic and simplest form of complex herbal formulations. In this study, we have examined the potential synergistic interactions of two botanical drugs in a specific ratio deriving from BHP of nine botanical drugs. Having previous studies of the multifunctional roles of DCS in neuroprotection and anti-inflammation (Ospondpant et al., 2022; Ospondpant et al., 2023), BHP of DCS with eight other botanical drugs was investigated. Firstly, our findings revealed that BHP of DCS and AEF at 1:1 w/w ratio demonstrated an optimal enhancement of activities in promoting neuronal differentiation and reducing Aβ fibril formation. Conversely, BHP of DCS and ZOR weakened these activities. In addition, the desertion of DCS and AEF from BHP of nine botanical drugs suggested that the other seven botanical drugs might be inactive and could not contribute to neuroprotection and anti-inflammation in this Thai traditional formulation. For further development of BHP, combining two botanical drugs, including DCS and AEF, was selected to determine the optimal composition. Among various tested ratios of BHP of DCS and AEF, a specific ratio of 1:9 w/w provided a strengthened stimulatory effect on neuronal differentiation. Notably, the effects of DCS:AEF (1:9) were greater than individual botanical drug extracts in terms of disrupting Aβ aggregation, preventing Aβ-induced cell toxicity, and suppressing LPS-induced inflammation. Similarly, the biological activities of PHP-6 were superior to loureirin A, loureirin B, resveratrol, pterostilbene, embelin, and syringic acid. While the present study provides valuable evidence of active phytochemicals within the BHP of DCS and AEF in cellular responses, there is a need to unravel the underlying mechanisms of action for the synergistic interaction between multiple components of this BHP. The application of network pharmacology in BHP can provide comprehensive insights into complex interactions between multiple active phytochemicals and their targets of action, which can support and further elucidate the synergistic interaction of BHP compositions (Panossian et al., 2024).

The current findings suggest that the six phytochemicals presented in DCS:AEF (1:9) might have the potential to represent, at least partly, the functionality of BHP. These phytochemicals could directly bind to Aβ peptides, thereby disrupting the formation of Aβ fibrils and aggregates. Indeed, the molecular docking analysis has demonstrated the possible binding ability of embelin to Aβ peptides (Bhuvanendran et al., 2019). Similar to curcumin, a well-known inhibitor of Aβ aggregation (Rao et al., 2015), embelin could directly bind to Aβ peptides, leading to the inhibition of Aβ fibrilization and conversion of Aβ into a non-toxic form. In addition, the docking analysis also indicated the possible bindings of pterostilbene and resveratrol to NGF (data not shown). This binding interaction could account for the induction of neurofilament expression that occurs when pterostilbene or resveratrol is presented in a low concentration of NGF. Our study suggests that the BHP of DCS and AEF, at a specific ratio of 1:9 w/w, could target potential therapeutic pathways, including Aβ peptide, which is believed to play a role in the pathogenesis of AD. The HPLC analysis revealed that embelin was a major compound presented in DCS:AEF (1:9) and AEF. These botanical drug extracts could directly inhibit the Aβ fibrilization, which was associated with an increase in cell survival. In addition, another potential therapeutic target involves the stimulation of NGF functions. In AD patients, the loss of cholinergic neurons is associated with memory impairment and cognitive decline. Maintaining the integrity and function of cholinergic neurons, as well as modulating neurogenesis and neurite outgrowth, are crucial roles of neurotrophic factors, such as NGF. The reduction of NGF levels has been detected in aging and individuals with AD, which contributes to the disruption of brain function (Hefti and Weiner, 1986; Connor and Dragunow, 1998). The combined effects of DCS:AEF (1:9) and a low dose of NGF had greater effects on the stimulation of neurite outgrowth and neuronal differentiation, as compared to individual treatment. Possibly, the active compounds of DCS:AEF (1:9), such as pterostilbene and resveratrol, exhibited the ability to bind to NGF, thereby enhancing the effects of NGF-induced neuronal differentiation. Similarly, the combination of peanut shell extract and a low dose of NGF was able to optimize the activities of neurite outgrowth and neuronal differentiation (Gao et al., 2022). This robust induction could be attributed to luteolin, a major flavonoid compound found in peanut shell extract, that showed the binding activity to NGF, leading to the enhancement of NGF activities. Indeed, peanut shell extract and luteolin could enhance NGF activities through the phosphorylations of tropomyosin receptor kinase A (TrkA), protein kinase B (Akt), extracellular signal-regulated kinase 1/2 (ERK1/2), and cAMP response element-binding protein (CREB) (Gao et al., 2021; Gao et al., 2022). Thus, the combination of DCS and AEF possibly has the potential to restore neuronal cell dysfunction in conditions characterized by insufficient levels of NGF during the development of AD. The third possible therapeutic target is the suppression of neuroinflammation. DCS:AEF (1:9) acts as an anti-inflammatory agent, reducing the expression of the proinflammatory molecules and promoting morphological changes in activated microglia to a resting stage. Previous study has reported that DCS could suppress the production of proinflammatory molecules through the phosphorylations of c-Jun N-terminal kinase (JNK), p38, and Akt in BV2 microglia, as well as decrease excessive phagocytosis of Aβ fibrils (Ospondpant et al., 2023).

The safety of botanical drug extracts is a concern. The current study revealed that the non-toxic concentration of ethanol extracts from DCS:AEF (1:9), DCS, and AEF at up to 10 μg/mL for BV2 microglial cells and 20 μg/mL for PC12 cells were identified. In addition, several animal studies have evaluated the safety and potential toxicity of these botanical drugs. The median lethal dose of intraperitoneally injected methanol and hexane extracts of DCS in mice was 1.67 g/kg and less than 739.73 mg/kg, respectively (Reanmongkol et al., 2003). The ethanol extract of AEF at 5 g/kg oral administration did not show any acute toxic effect or mortality in mice. In addition, the sub-chronic toxicity demonstrated that the administration of AEF at 20–2,000 mg/kg/day, for 90 consecutive days, did not have a significant impact on food consumption, organ weight, body growth, health status, or clinical values (Saktiyasunthorn et al., 2012).

Deriving from Thai traditional medicine, the advantages of having the BHP of DCS and AEF within the complex herbal formulation have been displayed in the functionalities of neuroprotection. This study provides valuable preliminary insight into the multifunctional roles of the BHP of DCS and AEF on cellular response; however, further validation using animal models and network pharmacology approaches is needed to understand their synergistic interaction. In conclusion, the findings suggest that the BHP of DCS and AEF might have promising applications as a neuroprotective agent and/or complementary therapeutic approach for neurodegenerative diseases, including AD.

## Data Availability

The original contributions presented in the study are included in the article/Supplementary Material, further inquiries can be directed to the corresponding author.

## Abbreviations

Aβ, amyloid β; AD, Alzheimer’s disease; AEF, *Ardisia elliptica* fruit; BHP, botanical hybrid preparation; CI, combination index; DCS, *Dracaena cochinchinensis* stemwood; HPLC, high-performance liquid chromatography; IL-1β, interleukin-1β; iNOS, inducible nitric oxide synthase; LPS, lipopolysaccharide; Luc, luciferase; MTT, 3-(4,5-dimethylthiazol)-2,5-diphenyl-tetrazolium bromide; NF, neurofilament; NGF, nerve growth factor; PHP, phytochemical hybrid preparation; RT-qPCR, real-time quantitative reverse transcription polymerase chain reaction; ThT, thioflavin T; TNF-α, tumor necrosis factor-α.

## References

[B1] Alzheimer’s Association (2022). 2022 Alzheimer's disease facts and figures. Alzheimers Dement. 18 (4), 700–789. 10.1002/alz.12638 35289055

[B2] Bhuvanendran S. Hanapi N. A. Ahemad N. Othman I. Yusof S. R. Shaikh M. F. (2019). Embelin, a potent molecule for Alzheimer's disease: a proof of concept from blood-brain barrier permeability, acetylcholinesterase inhibition and molecular docking studies. Front. Neurosci. 13, 495. 10.3389/fnins.2019.00495 31156375 PMC6532548

[B3] Bureau of Drug and Narcotic (2021). Thai herbal pharmacopoeia 2021. Thailand: Ministry of Public Health.

[B4] Caesar L. K. Cech N. B. (2019). Synergy and antagonism in natural product extracts: when 1 + 1 does not equal 2. Nat. Prod. Rep. 36 (6), 869–888. 10.1039/c9np00011a 31187844 PMC6820002

[B5] Che C. T. Wang Z. J. Chow M. S. Lam C. W. (2013). Herb-herb combination for therapeutic enhancement and advancement: theory, practice and future perspectives. Molecules 18 (5), 5125–5141. 10.3390/molecules18055125 23644978 PMC6269890

[B6] Chou T. C. (2006). Theoretical basis, experimental design, and computerized simulation of synergism and antagonism in drug combination studies. Pharmacol. Rev. 58 (3), 621–681. 10.1124/pr.58.3.10 16968952

[B7] Committee on Document and Archive Preparation (2009). “Committee on document and archive preparation under the steering committee on organizing the events for honouring his majesty the king, published to commemorate HM the king’s 6^th^ cycle or 72^nd^ birthday anniversary, 5 december 1999,” in Royal medical textbook (tamra vejjasart chabab luang) of king Rama V, volumes 1 and 2.

[B8] Connor B. Dragunow M. (1998). The role of neuronal growth factors in neurodegenerative disorders of the human brain. Brain Res. Rev. 27 (1), 1–39. 10.1016/s0165-0173(98)00004-6 9639663

[B9] Cummings J. L. Tong G. Ballard C. (2019). Treatment combinations for Alzheimer's disease: current and future pharmacotherapy options. J. Alzheimers Dis. 67 (3), 779–794. 10.3233/JAD-180766 30689575 PMC6398562

[B10] Gao A. X. Xia T. C. Shing-Hung Mak M. Kin-Leung Kwan K. Zhong-Yu Zheng B. Xiao J. (2021). Luteolin stimulates the NGF-induced neurite outgrowth in cultured PC12 cells through binding with NGF and potentiating its receptor signaling. Food Funct. 12 (22), 11515–11525. 10.1039/d1fo01096d 34704574

[B11] Gao A. X. Xiao J. Xia T. C. Dong T. T. Tsim K. W. (2022). The extract of peanut shell enhances neurite outgrowth of neuronal cells: recycling of agricultural waste for development of nutraceutical products. J. Funct. Foods 91, 105023. 10.1016/j.jff.2022.105023

[B12] Hefti F. Weiner W. J. (1986). Nerve growth factor and Alzheimer's disease. Ann. Neurol. 20 (3), 275–281. 10.1002/ana.410200302 3532929

[B13] Li Y. R. Li S. Lin C. C. (2018). Effect of resveratrol and pterostilbene on aging and longevity. Biofactors 44 (1), 69–82. 10.1002/biof.1400 29210129

[B14] Mulholland J. (1979). Thai traditional medicine: ancient thought and practice in a Thai context. J. Siam Soc. 67 (2), 80–115. 11617470

[B15] Ondee S. Sithisarn P. Mangmool S. Rojsanga P. (2020). Chemical standardization and anti-proliferative activity of *Ardisia elliptica* fruit against the HCT116 human colon cancer cell line. Molecules 25 (5), 1023. 10.3390/molecules25051023 32106546 PMC7179138

[B16] Ospondpant D. Gao X. Dong T. T. Tsim K. W. K. (2022). *Dracaena cochinchinensis* stemwood extracts inhibit amyloid-β fibril formation and promote neuronal cell differentiation. Front. Pharmacol. 13, 943638. 10.3389/fphar.2022.943638 36147317 PMC9486383

[B17] Ospondpant D. Xia Y. Lai Q. W. S. Yuen G. K. Yang M. Chanthanam K. (2023). The extracts of *Dracaena cochinchinensis* stemwood suppress inflammatory response and phagocytosis in lipopolysaccharide-activated microglial cells. Phytomedicine 118, 154936. 10.1016/j.phymed.2023.154936 37385071

[B18] Panossian A. Lemerond T. Efferth T. (2024). State-of-the-art review on botanical hybrid preparations in phytomedicine and phytotherapy research: background and perspectives. Pharmaceuticals 17 (4), 483. 10.3390/ph17040483 38675443 PMC11053582

[B19] Perry E. Howes M. J. (2011). Medicinal plants and dementia therapy: herbal hopes for brain aging? CNS Neurosci. Ther. 17 (6), 683–698. 10.1111/j.1755-5949.2010.00202.x 22070157 PMC6493900

[B20] Rao P. P. Mohamed T. Teckwani K. Tin G. (2015). Curcumin binding to beta amyloid: a computational study. Chem. Biol. Drug Des. 86 (4), 813–820. 10.1111/cbdd.12552 25776887

[B21] Reanmongkol W. Subhadhirasakul S. Bouking P. (2003). Antinociceptive and antipyretic activities of extracts and fractions from *Dracaena loureiri* in experimental animals. Songklanakarin J. Sci. Technol. 25 (4), 467–476.

[B22] Saktiyasunthorn N. Chivapat S. Sincharoenpokai P. Rungsipipat A. Maneechai N. Suphaphon B. (2012). Acute and subchronic toxicity study of *Ardisia ellipica* Thunb. fruit extract. Thai J. Vet. Med. 42 (2), 201–207. 10.56808/2985-1130.2383

[B23] Srinivasulu C. Ramgopal M. Ramanjaneyulu G. Anuradha C. M. Suresh Kumar C. (2018). Syringic acid (SA) - a review of its occurrence, biosynthesis, pharmacological and industrial importance. Biomed. Pharmacother. 108, 547–557. 10.1016/j.biopha.2018.09.069 30243088

[B24] World Health Organization (2017). Global action plan on the public health response to dementia 2017-2025. Available at: https://www.who.int/publications/i/item/global-action-plan-on-the-public-health-response-to-dementia-2017---2025 (Accessed November 28, 2023).

[B25] Xue C. Lin T. Y. Chang D. Guo Z. (2017). Thioflavin T as an amyloid dye: fibril quantification, optimal concentration and effect on aggregation. R. Soc. Open Sci. 4 (1), 160696. 10.1098/rsos.160696 28280572 PMC5319338

[B26] Yuan A. Nixon R. A. (2021). Neurofilament proteins as biomarkers to monitor neurological diseases and the efficacy of therapies. Front. Neurosci. 15, 689938. 10.3389/fnins.2021.689938 34646114 PMC8503617

[B27] Zhou X. Seto S. W. Chang D. Kiat H. Razmovski-Naumovski V. Chan K. (2016). Synergistic effects of Chinese herbal medicine: a comprehensive review of methodology and current Research. Front. Pharmacol. 7, 201. 10.3389/fphar.2016.00201 27462269 PMC4940614

