# Supplementary material for: Synergy of botanical drug extracts from Dracaena cochinchinensis stemwood and Ardisia elliptica fruit in multifunctional effects on neuroprotection and anti-inflammation
Source: Front Pharmacol. 2024 May 1;15:1399549. doi: 10.3389/fphar.2024.1399549 (PMC11094215; doi:10.3389/fphar.2024.1399549)
Supplement: Supplementary file 1 [file DataSheet1.pdf]

Supplementary Material

Supplementary Table 1. HPLC analysis of ethanol extracts of DCS:AEF (1:9 w/w), DCS, and AEF

Extracts	Yield (%)[a]	Identified compounds	Content (μg/mg)[b]
**DCS:AEF (1:9)**	7.08 ± 0.66	Syringic acid	3.84 ± 0.68
		Resveratrol	2.30 ± 0.02
		Loureirin A	2.78 ± 0.30
		Loureirin B	3.22 ± 0.18
		Pterostilbene	5.56 ± 0.58
		Embelin	73.08 ± 7.89
**DCS**	20.58 ± 2.09	Resveratrol	3.98 ± 0.85
		Loureirin A	5.37 ± 0.74
		Loureirin B	5.89 ± 0.85
		Pterostilbene	16.52 ± 4.27
**AEF**	6.17 ± 0.33	Syringic acid	6.99 ± 0.91
		Embelin	72.57 ± 2.53

The contents of identified compounds containing in herbal extracts (10 mg) were analyzed by HPLC-DAD with a TC-C18 column (4.6 x 250 mm, 5 μm). The peak identities were indicated in Sup Figure 1.

[a] The percent yields per dried weight of herbal extracts in mean ± SEM, *n* = 3.

[b] The values are measured from HPLC analysis in mean ± SEM, *n* = 3.

Supplementary Table 2. The primer sequences were performed using RT-qPCR analysis

Genes	Species	Forward primer (5′-3′)	Reverse primer (5′-3′)
Nefm	Rat	CTA AGG AGT CCC TGG AAC GGC	TCC CAC TTT GTT CCC CGA AGC
GAPDH	Rat	CTT CTT GTG CAG TGC CAG CC	CAG CCT TGA CTG TGC CGT TG
IL-1β	Mouse	GTG GTA TTC TCC ATG AGC TT	TTC ATC ACA CAG GAC AGG TA
TNF-α	Mouse	AGT GAC AAG CCT GTA GCC	AGG TTG ACT TTC TCC TGG
iNOS	Mouse	ACG AGA CGG ATA GGC AGA GA	CAC ATG CAA GGA AGG GAA CT
GAPDH	Mouse	AAC GGA TTT GGC CGT ATT GG	CTT CCC GTT CAG CTC TGG G

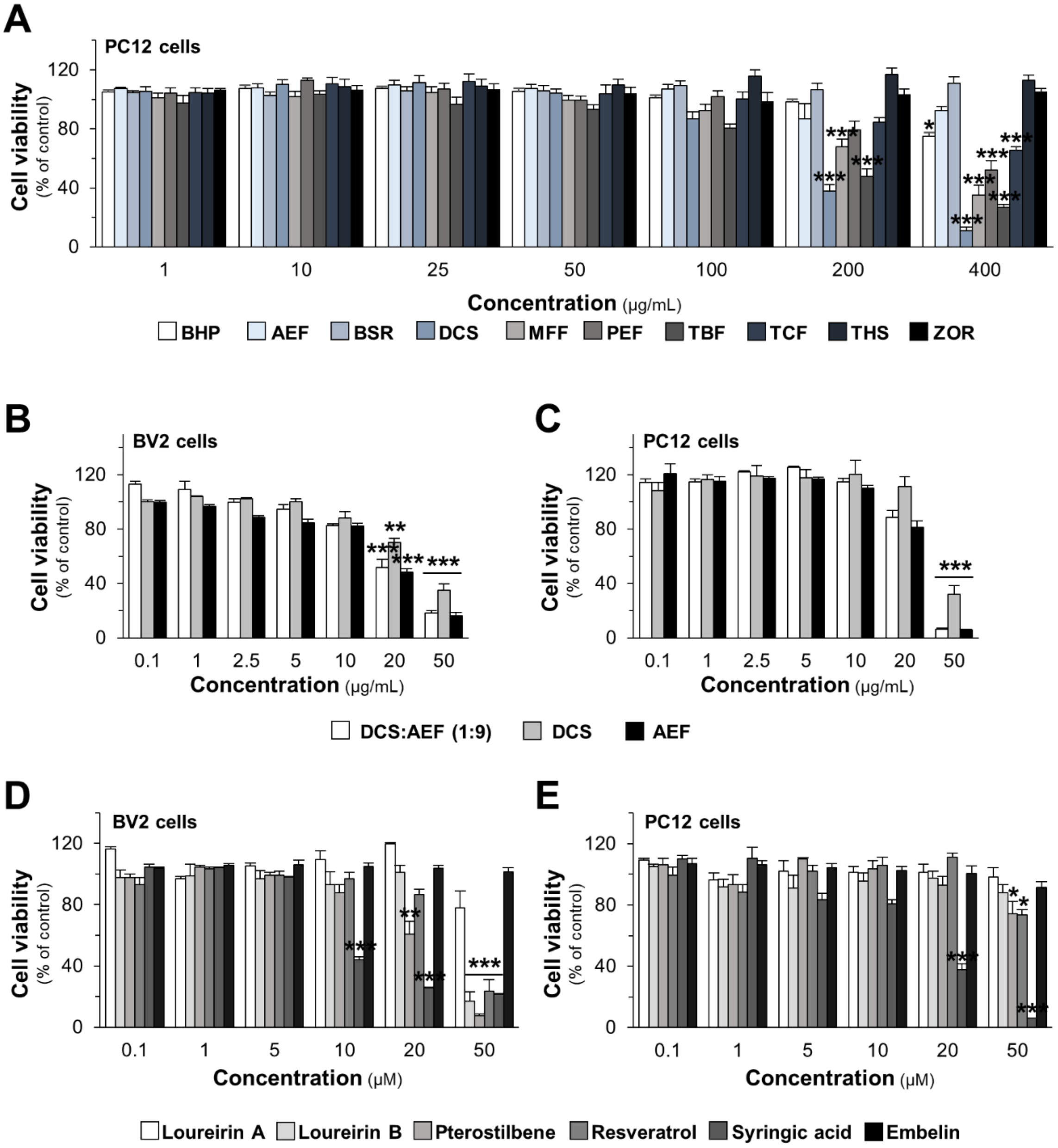

Supplementary Figure 1. Cytotoxicity of botanical drug extracts and phytochemicals

(A) PC12 cells were treated with aqueous extracts from botanical hybrid preparation (BHP) of nine botanical drugs and single botanical drugs for 48 h. BV2 cells were treated with **(B)** ethanol extracts, including DCS:AEF (1:9), DCS, and AEF, or **(D)** single compounds, including loureirin A, loureirin B, pterostilbene, resveratrol, syringic acid, and embelin, for 24 h. PC12 cells were treated with **(C)** ethanol extracts from botanical drug extracts or **(E)** single compounds for 48 h. The cell viability was determined using the MTT assay. Data are shown in mean ± SEM of the percentage of control ($n = 4$). *$p < 0.05$, **$p < 0.01$, and ***$p < 0.001$ as compared to untreated cells (control).
